# Associations Between PTSD Symptom Custers and Longitudinal Changes in Suicidal Ideation: Comparison Between 4-Factor and 7-Factor Models of DSM-5 PTSD Symptoms

**DOI:** 10.3389/fpsyt.2021.680434

**Published:** 2021-11-16

**Authors:** Che-Sheng Chu, Po-Han Chou, Shao-Cheng Wang, Masaru Horikoshi, Masaya Ito

**Affiliations:** ^1^Department of Psychiatry, Kaohsiung Veterans General Hospital, Kaohsiung, Taiwan; ^2^Center for Geriatrics and Gerontology, Kaohsiung Veterans General Hospital, Kaohsiung, Taiwan; ^3^Graduate Institute of Medicine, College of Medicine, Kaohsiung Medical University, Kaohsiung, Taiwan; ^4^Department of Psychiatry, China Medical University Hsinchu Hospital, China Medical University, Hsinchu, Taiwan; ^5^Department of Psychiatry, China Medical University Hospital, China Medical University, Taichung, Taiwan; ^6^Department of Biological Science and Technology, National Chiao Tung University, Hsinchu, Taiwan; ^7^Biological Optimal Imaging Lab, Department of Photonics, College of Electrical and Computer Engineering, National Chiao Tung University, Hsinchu, Taiwan; ^8^Department of Psychiatry, Taoyuan General Hospital, Ministry of Health and Welfare, Taoyuan, Taiwan; ^9^Department of Mental Health, Johns Hopkins Bloomberg School of Public Health, Baltimore, MD, United States; ^10^Department of Medical Laboratory Science and Biotechnology, Chung Hwa University of Medical Technology, Tainan, Taiwan; ^11^National Center for Cognitive-Behavior Therapy and Research, National Center of Neurology and Psychiatry, Tokyo, Japan

**Keywords:** posttraumatic stress disorder, suicide ideation, DSM, symptomatology, anhedonia, externalizing behavior, PTSD

## Abstract

**Objective:** The association between posttraumatic stress disorder (PTSD) and suicidal ideation (SI) is well-known. However, a few studies have investigated the associations between PTSD symptom clusters based on the fifth edition of the Diagnostic and Statistical Manual for Mental Disorders (DSM-5) and changes in suicide risk longitudinally.

**Methods:** We adopted a longitudinal study design using data from the National Survey for Stress and Health of 3,090 of the Japanese population. The first and second surveys were conducted on November 2016 and March 2017, respectively. The suicidal ideation attributes scale was applied to assess the severity of suicidal ideation at baseline and the follow-up period. A multivariate linear regression model was conducted to examine the associations between the 4- or 7-factor model of PTSD symptom clusters at baseline and longitudinal changes in SI.

**Results:** Overall, 3,090 subjects were analyzed (mean age, 44.9 ± 10.9 years; 48.8% female) at Baseline, and 2,163 completed the second survey. In the 4-factor model, we found that the severity of negative alternations in cognition and mood were significantly associated with increased SI after 4 months. In the 7-factor model, we found that the severity of anhedonia and externalizing behavior at baseline was significantly associated with increased SI during the follow-up period.

**Conclusions:** We found that the seven-factor model of DSM-5 PTSD symptoms may provide greater specificity in predicting longitudinal SI change in the general population. Closely monitoring specific PTSD core symptoms may be more effective in mitigating key clinical and functional outcomes.

## Introduction

According to the World Health Organization report, the crude suicide rate is estimated at 9.0 per 100,000 of the populations worldwide, with the highest at 16.1 in the United States of America (USA) (1). Suicide takes a tremendous emotional and economic burden for individuals, families, and communities. In 2013, ~93.5 billion was the national cost of suicide, and suicide attempts reported in the USA. Lost productivity represents most of the cost (2). Therefore, early detection and management of suicide are vital, especially for those who have risk factors.

Emerging evidence has demonstrated that posttraumatic stress disorder (PTSD) can be an independent predictor of suicide attempt (3). In a nationwide study of 3.1 million people in Sweden, individuals diagnosed with PTSD were twice more likely to die by suicide than those without PTSD (4). A meta-analysis showed that PTSD is strongly associated with increased suicidality, including suicidal thoughts, behaviors, plans, attempts, and suicides (5). PTSD has been reorganized into four clusters: intrusion symptoms, avoidance, negative alternations in cognition and mood (NCAM), and alterations in arousal and reactivity (AAR) based on the Diagnostic and Statistical Manual of Mental Disorder (DSM-5) (6). The PTSD checklist (PCL), a brief and self-reported instrument, is mostly used to quantify and monitor symptoms of PTSD over time, and to screen individuals for PTSD in both research and clinical settings (7). The PCL, which was recently updated to PCL-5, includes 20 items that correspond to the 20 PTSD symptoms in accordance with DSM-5 (8). PCL-5 appears to have excellent temporal stability and yields PTSD prevalence estimates similar to that of the previous version (9). However, recently, several confirmatory factor analysis (CFA) studies, in contrast to the four-factor model, have found that the best-fitting model is a seven-factor model, which includes intrusion symptoms, avoidance, negative affect, anhedonia, externalizing behaviors, anxious arousal, and dysphoric arousal (10–13).

A growing amount of evidence has demonstrated a positive relationship between specific PTSD symptoms and suicide risk or suicidal ideation (SI) (14). However, evidence is equivocal regarding the specific PTSD symptom clusters that may confer SI *via* DSM-IV and DSM-5. For example, some studies have linked hyperarousal (15, 16) or re-experiencing (14, 17–19) to increased SI, whereas others have linked numbing (16, 18), avoidance (20), and dysphoria (21) to elevated SI. Although the results of specific symptoms linked to SI have been inconsistent, overall evidence shows a positive relationship between at least one PTSD cluster and SI. Explanations for the inconsistent findings might be derived from cross-sectional study design, small sample sizes, the enrollment of specific occupation population such as military personnel (16), veterans (17), or firefighters (18), and even ethnic differences (22); therefore, the findings may have been confounded by selection bias.

To the best of our knowledge, there have been no studies to date that have examined the associations between PTSD symptoms and longitudinal changes in the risk of SI using a nationwide survey database. The aim of our study was to examine the associations between specific PTSD symptom clusters in four- and seven-factorial models and longitudinal SI changes in a sample of the Japanese population.

## Methods

### Database

Data were obtained from the National Survey for Stress and Health (NSSH), which was conducted between 2016 and 2017. A flow chart of the survey is shown in Supplementary Figure 1. To control the seasonal effect, two waves of the survey were conducted. In Wave 1, participants were screened since November 2016, and the survey was administered twice (Time 1 in November 2016 and Time 2 in March 2017). Wave 2 consisted of screening and a Time 1 survey (both in March 2017). The questions used in the screen and the Time 1 surveys were the same across Waves 1 and 2, with a random scale order in Wave 2 only. We sent an advertising email to 100,077 panelists in November (Wave 1) and to 56,953 panelists in March (Wave 2). Of these, 20,000 participated in each screen on November 15–30, 2016 (Wave 1) and March 23–25, 2017 (Wave 2). At the screening, participants were identified their index traumatic event at the beginning of the questionnaire packet. Participants who reported no traumatic events were asked to identify their most stressful or heartbreaking life event. The identified index event automatically appeared in the instructions for answering all subsequent PCL-5 or other PTSD-related assessments. In the screen, participants answered the PCL-5 and questions regarding the status of the diagnosis and treatment of PTSD. Our target sample size was 6,000 individuals, including 3,000 patients with PTSD who met the probable DSM-5 diagnostic criteria using the PCL-5, 1,000 nonclinical panelists without any traumatic experience, and 2,000 nonclinical or subclinical panelists with traumatic experiences. We terminated the screen in Waves 1 and 2 upon reaching half of the target sample size. The screened participants answered measures of psychiatric symptoms and psychological processes at Times 1 and 2 for the purposes of examining the various study hypotheses beyond this report. Only Wave 1 participants took part in the Time 2 survey 4 months after Time 1. Detailed information regarding the NSSH could be found in our previous work (11, 19).

All participants read a full explanation of the study and gave informed consent before answering the questionnaires during the screen and the Time 1 and Time 2 surveys. All survey content was examined for logical flow, face validity, design, and miscellaneous errors by nine clinical psychologists and was double-checked by two Macromill survey engineers. To enhance data quality, the survey system automatically excluded participants who showed extremely short response times. Because the questionnaire did not allow participants to proceed with unanswered items, no data were missing except for income. The institutional review board at the National Center of Neurology and Psychiatry approved the study (Approval Number: A2015-086). We conducted the study with a longitudinal design and used the data collected at Wave 1 (*n* = 3,090). The measurements at Time 1 and Time 2 surveys are shown in Supplementary Figure 2.

### Demographics

Personal information including sex, age, marital status, income, history of physical or psychological abuse, history of habitual use of alcohol/tobacco, history of self-harm behavior, history of being diagnosed and treated for any mental disorder including major depressive disorder (MDD), obsessive–compulsive disorder (OCD), seasonal affective disorder (SAD), panic disorder (PAD), psychotic disorder, bipolar disorder, PTSD, eating disorder, generalized anxiety disorder (GAD), and dysthymic disorder were included (Table 1).

**Table 1:** Characteristics of the study participants.

**Characteristic**	**Participants of Wave 1**	**Wave 1 participant at follow-ups**
	*N* = 3,090	*N* = 2,163
**Female**	1,509 (48.8%)	990 (45.8%)
**Age (mean** **±** **SD)**		
	44.9 ± 10.9	46 ± 10.7
**Marital status**		
Married	1,466 (47.4%)	1,038 (48.0%)
**Personal yearly income (yen)**		
0–1,999,999	1,460 (54.3%)	1,019 (49.7%)
2,000,000–3,999,999	572 (21.3%)	420 (20.5%)
4,000,000–5,999,999	320 (11.9%)	237 (11.6%)
6,000,000–7,999,999	197 (7.3%)	140 (6.8%)
8,000,000–9,999,999	80 (3.0%)	60 (2.9%)
Over 10,000,000	40 (1.5%)	36 (1.7%)
**Hx of physical abuse**	1,222 (39.6%)	818 (37.8%)
**Hx of emotional abuse**	1,875 (60.7%)	1,284 (59.4%)
**Habitual use of alcohol**	1,211 (39.2%)	849 (39.3%)
**Habitual use of tobacco**	920 (29.8%)	669 (30.9%)
**Hx of self-harm**	778 (25.2%)	539 (24.9%)
**Diagnostic and Statistical Manual for Mental Disorders (DSM-5) diagnostic status based on PCL-5**		
Posttraumatic stress disorder (PTSD)	1,545 (50%)	1,005 (46.5%)
Trauma experienced without PTSD	930 (30.1%)	716 (33.1%)
No trauma experienced	515 (16.7%)	369 (17.1%)
Acute stress disorder (ASD)	44 (1.42%)	32 (1.5%)
Trauma experienced within 1 month but no ASD	56 (1.81%)	41 (1.9%)
**Psychiatric comorbidities diagnosed and treated in medical settings**		
Major depressive disorder (MDD)	1„630 (52.8%)	1159 (53.6%)
Obsessive–compulsive disorder (OCD)	471 (15.2%)	332 (15.4%)
Seasonal affective disorder (SAD)	489 (15.8%)	350 (16.2%)
Panic disorder	630 (20.4%)	434 (20.0%)
Psychosis	321 (10.4%)	210 (9.7%)
Bipolar disorder	335 (10.8%)	232 (10.7%)
PTSD	422 (13.7%)	289 (13.4%)
Eating disorder	293 (9.5%)	202 (9.3%)
Generalized anxiety disorder (GAD)	487 (15.8%)	350 (16.2%)
Dysthymic disorder	341 (11.0%)	234 (10.8%)
**Suicidal Ideation Attributes Scale SIDAS score at Time 1**	20.0 ± 9.7	19.8 ± 9.5
**SIDAS score at Time 2**		19.6 ± 9.1

*ASD, acute stress disorder; DSM, diagnostic and statistical manual for mental disorders; GAD, generalized anxiety disorder; MDD, major depressive disorder; OCD, obsessive compulsive disorder; PCL, the PTSD checklist; PTSD, post-traumatic stress disorder; SAD, seasonal affective disorder; SD, standard deviation; SIDAS, Suicidal Ideation Attributes Scale*.

### Measures

#### PCL-5

We used the Japanese version of the PCL-5 to assess PTSD symptoms (11). The PCL-5 included a 20-item assessment (8). The 20 items, which were concordant with the DSM-5 diagnostic items, were answered on a five-point Likert scale (0 = Not at all, 1 = A little bit, 2 = Moderately, 3 = Quite a bit, and 4 = Extremely). The item mapping for the four- and seven-factorial models are shown in Table 2.

**Table 2:** Item mapping for different factorial models for the PCL.

**Item**	**Item description**	**DSM-IV**	**DSM-5 4-factor**	**DSM-5 7-factor**
1 (B1)	Memories	R	R	R
2 (B2)	Dreams	R	R	R
3 (B3)	Flashbacks	R	R	R
4 (B4)	Cued distress	R	R	R
5 (B5)	Cued physical reactions	R	R	R
6 (C1)	Avoiding internal reminders	A/N	AV	AV
7 (C2)	Avoiding external reminders	A/N	AV	AV
8 (D1)	Dissociative amnesia	A/N	NAMC	NA
9 (D2)	Negative beliefs	A/N	NAMC	NA
10 (D3)	Blame	A/N	NAMC	NA
11 (D4)	Negative feelings	A/N	NAMC	NA
12 (D5)	Loss of interest	A/N	NAMC	An
13 (D6)	Detachment or estrangement	A/N	NAMC	An
14 (D7)	Numbing	A/N	NAMC	An
15 (E1)	Irritability or aggressive behavior	H	H	EB
16 (E2)	Reckless behavior	H	H	EB
17 (E3)	Hypervigilence	H	H	AA
18 (E4)	Startle	H	H	AA
19 (E5)	Concentration	H	H	DA
20 (E6)	Sleep	H	H	DA

*DSM-5, Diagnostic and Statistical Manual of Mental Disorders (5th Edition), R, re-experiencing, AV, avoidance, NAMC, negative alterations in, cognition and mood, H, hyperarousal, EB, externalizing behavior, AA. anxious arousal, DA. dysphoric arousal, An: anhedonia, NA. negative affect. A/N, avoidance/numbing*.

#### Traumatic Events

We used the 25 traumatic experience items in the PTSD module of the World Health Organization Composite International Diagnostic Interview, version 3.0 (WHO-CIDI) (23). If a participant answered 0 (never experienced) on all the 25 items, the participant was asked to recall the most stressful and heartbreaking event in their life was and then answered the items in the questionnaire. We used a four-point scale to assess the lifetime experiences of the participants (0, “never experienced”; 1, “experienced within the last month”; 2, “experienced once more than 1 month ago”; and 3, “experienced at least twice more than 1 month ago”). After completing the list of traumatic events, participants were asked to select one item from which they replied a 1, 2, or 3 as the worst that they had experienced.

#### Suicidal Ideation Attributes Scale

The Suicidal Ideation Attributes Scale (SIDAS) assesses the severity of suicidal ideation during the past month. Its five items ask about frequency, controllability, closeness to attempt, level of distress associated with the thoughts, and impact on daily functioning (24). Answers are reported on an 11-point Likert scale. The SIDAS exhibits high internal consistency (Cronbach's alpha = 0.91). Scores ≥ 21 indicated a high risk of suicide ideation (24).

### Statistical Analysis

For sensitivity analysis, Student's *t*-tests and chi-square were used to compare the characteristics between those who were lost to follow-ups and under follow-ups at Time 2. Multivariate linear regression models with longitudinal changes in SIDAS scores as the dependent variable and PTSD symptom clusters based on the four- or seven-factor model as the independent variables were adjusted for sociodemographic variables, history of self-harm, history of physical and psychological abuse, psychiatric comorbidities, and baseline SIDAS score as confounding factors. The multivariate linear regression models calculated the beta coefficients and found a 95% confidence interval. A *p*-value of < 0.05 indicated statistical significance, and all results were analyzed using STATA version 15.1.

## Results

### Clinical Characteristics of the Study Population

The baseline sociodemographic characteristics are shown in Table 1. Data from a total of 3,090 subjects were analyzed (mean age, 44.9 ± 10.9 years; 48.8% female) at Time 1 of Wave 1, and 2,163 subjects completed the survey at Time 2. There was no significant difference in demographic characteristics between those who were lost to follow-ups and under follow-ups at Time 2 in the sensitivity analysis. A DSM-5 diagnostic status based on the PCL-5 was obtained and showed that PTSD (50%) was the most common diagnosis, followed by trauma experienced without PTSD (30.1%), and no trauma experienced (16.7%). The most common traumatic experiences were emotional abuse (60.7%, *n* = 1,875), followed by physical violence (39.6%, *n* = 1,222). Among the recruited subjects, the most common psychiatric comorbidity was MDD (52.8%), followed by panic disorder (20.4%), SAD (15.8%), and GAD (15.8%). The mean baseline SIDAS scores were 20.0 ± 9.7 at Time 1.

### Risk Factors Associated With Posttraumatic Stress Disorder Symptoms and Change in Suicidal Ideation in the Four-Factor Model

After multiple linear regression analysis (adjusted *R*^2^ = 0.313), we found an history of physical abuse [beta = 1.16, 95% confidence interval (95% CI) = 0.34–1.97, and *p* = 0.005], previous suicide attempt (beta = 0.78, 95% CI = 0.40–1.17, and *p* < 0.001), comorbid with diagnosis of MDD (beta = 0.74, 95% CI = 0.44–1.43, and *p* = 0.037), and NACM symptoms based on the DSM-5 four-factor model (beta = 0.12, 95% CI = 0.02–0.22, and *p* = 0.016) were significantly associated with an increased SIDAS score (Table 3).

**Table 3:** Results of linear regression for the associations between PTSD symptom clusters and suicide risk in different models.

**Model of PCL-5 PTSD symptom clusters**	**ß**	**95% CI**	***p-*value**
**4-Factor model**
Re-experiencing	0.07	−0.04−0.19	0.203
Avoidance	−0.18	−0.39−0.03	0.097
Negative alterations in cognition and mood^*^	0.12	0.02−0.22	0.016
Hyperarousal	0.11	−0.01−0.22	0.066
**7-Factor model**
Re-experiencing	0.08	−0.04−0.19	0.176
Avoidance	−0.12	−0.34−0.09	0.272
Negative affect	−0.01	−0.17−0.16	0.958
Anhedonia^*^	0.25	0.07−0.43	0.008
Externalizing behavior^*^	0.33	0.09−0.57	0.007
Anxious arousal	−0.08	−0.32−0.16	0.500
Dysphoric arousal	0.09	−0.13−0.32	0.411

* *Statistically significant difference. CI, confidence interval; PCL, the PTSD checklist; PTSD, posttraumatic stress disorder*.

### Risk Factors Associated With Posttraumatic Stress Disorder Symptoms and Longitudinal Change in Suicidal Ideation in the Seven-Factor Model

In the seven-factor model (adjusted *R*^2^ = 0.315), history of physical abuse (beta = 1.19, 95% CI = 0.38–2.01, and *p* = 0.004), previous suicide attempt (beta = 0.74, 95% CI = 0.35–1.13, and *p* < 0.001), comorbid with diagnosis of OCD (beta = 1.17, 95% CI = 0.03–2.32, and *p* = 0.045), PTSD symptom clusters of anhedonia (beta = 0.25, 95% CI = 0.07–0.43, and *p* = 0.008), and externalizing behavior (beta = 0.33, 95% CI = 0.09–0.57, and *p* = 0.007) were significantly associated with increased SIDAS score (Table 3).

## Discussion

To our knowledge, the is the first study investigating the associations between PTSD symptom clusters and longitudinal change in SI using a nationwide survey dataset. In the four-factor model, we found that history of physical abuse, previous suicide attempt, comorbid with diagnosis of MDD, and NACM symptom clusters were associated with higher risk of increasing SI. In the seven-factor model, we found that history of physical abuse, previous suicide attempt, comorbid with diagnosis of OCD, and anhedonia and externalizing behavior were associated with higher risk of increasing SI.

### Comparison Between the Four- and Seven-Factor Model of Posttraumatic Stress Disorder Symptoms in PCL-5

In both the four- and seven-factor model of PTSD symptoms, we found that physical abuse, previous suicide attempt, and comorbid with diagnosis of MDD or OCD were significant predictors for future suicide risk. These are well-known risk factors and are consistent with previous studies (25, 26). For psychometric properties of the PCL-5, we found more significant and specific clusters of symptoms predicting longitudinal changes in SI in the seven-factor model compared with the four-factor model. So far, no such study has compared the ability of the four- and 7-factor model to predict future SI among people who have experienced various traumatic event types. Results of this study extend previous CFA studies of DSM-5 PTSD, indicating that a seven-factor hybrid model of DSM-5 PTSD symptom clusters provides greater specificity in understanding associations with comorbid psychopathology and suicidal ideation (10–14).

### Specific Posttraumatic Stress Disorder Symptom Clusters of the Four-Factor Model Predict Change in Suicidal Ideation

The present longitudinal follow-up study is the first to report NACM as a predictor for future SI change. A few studies have examined the association between this factor and SI, as the NACM factor was newly introduced with the release of the DSM-5. Similar to previous studies, NACM symptoms were associated with increased SI (16, 18, 19, 21, 27). In a study conducted by Brown et al., all PTSD factors were positively correlated with SI, and NACM and AAR had the strongest associations (27). Another study demonstrated that the NACM cluster, anhedonia, and negative beliefs based on symptom-level analysis were most strongly correlated with a history of suicide attempts (28). NACM symptoms are conceptualized as being distress based and hypothesized to co-occur with PTSD and depression, as supported by comorbidity with MDD predicting SI change in our study (29). The reason for NACM prediction of SI change might be explained at the symptom level. NACM consists of negative beliefs, blame, loss of interest, social detachment, and numbing, which were highly correlated with SI. For example, a systemic review study has shown that social detachment, especially subjective feeling of loneliness, is strongly associated with suicide risk (30).

### Specific Posttraumatic Stress Disorder Symptom Clusters of the Seven-Factor Model to Predict Changes in Suicidal Ideation

In the seven-factor model, previous studies have found that hyperarousal (15, 16), re-experiencing (14, 17–19), anhedonia (19, 31), and externalizing behavior (19, 31) were associated with increased SI. The present study found that anhedonia and externalizing behavior were strong predictors of future worsening SI. Anhedonia symptom could be considered as a core construct in contemporary transdiagnostic models of psychopathology (31, 32). Anhedonia is a common feature following exposure to traumatic stress and, thus, as a potential predictor for disease course. Furthermore, a new externalizing behavior factor, which combined irritability or aggressive behavior with reckless behavior, was another significant predictor for suicidal outcome. The externalizing behaviors reflect the difficulties with impulse control and emotional regulation (33). Taken together, both subjective trauma-related symptoms (e.g., anhedonia) and objective externalizing behaviors (e.g., risk-taking behaviors) may have value in predicting suicide risk in the trauma-exposed general population. However, the present study consisted of a representative cohort of the Japanese general population and was the first to use the seven-factor model of DSM-5 PTSD symptoms to predict SI change. Therefore, future studies enrolling different populations (such as veterans or clinic-based settings) from different countries are warranted to confirm our findings.

### Clinical Implications

The present study has important clinical implications for clinical practice and for the basic research of PTSD phenomenology. First, our study suggested that a seven-factor model of DSM-5 PTSD symptoms provides greater specificity than a four-factor model in predicting future longitudinal changes in SI. Second, patients who experienced trauma with symptoms of anhedonia and externalizing behaviors represent a highly risky population for suicide and warrants careful evaluation. Clinicians should be cautious about conducting comprehensive suicide risk assessments and to engage in brief suicide mitigation interventions to prevent unwanted accidents.

### Limitations and Strengths

Several limitations of the study should be addressed. First, the self-report online instruments were adopted to evaluate the PTSD symptoms and clinical outcomes. The possibility of increasing endorsements of sensitive responses of online surveys because of anonymity should be considered (27). Second, the participants were limited to those who had internet access and registered as panelists for the survey company. Third, the subjects of the study were Japanese. Hence, it is hard to generalize the present findings to different ethnic groups. Fourth, some subjects replied no experiences of traumatic events but have symptoms of PTSD, and we cannot exclude the possibility that some participants may misunderstand the instruction as this survey was conducted online.

Despite the limitations, a notable strength of the present study was to include the large Japanese population using DSM-5-based PTSD symptoms. Additionally, this is the first study to compare the four- and seven-factor models of PTSD symptoms to predict longitudinal changes in SI.

## Conclusion

Our study found that some factors of the seven-factor model might be more useful to predict suicidality and might, therefore, have more value than the four-factor model factors. In the seven-factor model, symptoms specifically for anhedonia and externalizing behaviors may be meaningful predictors for future SI worsening in the general population.

Close monitoring of these core PTSD symptoms and implementing established treatments are vital to prevent further accidents in the general population.

## Data Availability Statement

The raw data supporting the conclusions of this article will be made available by the authors, without undue reservation.

## Ethics Statement

The studies involving human participants were reviewed and approved by the institutional review board at the National Center of Neurology and Psychiatry, Japan. The patients/participants provided their written informed consent to participate in this study.

## Author Contributions

MI conceptualized the study and took charge of the project administration. P-HC, S-CW, and MI formulated the methodology and validated the study. P-HC and S-CW performed the formal analysis. C-SC, P-HC, and S-CW wrote the original draft. C-SC, P-HC, S-CW, and MH reviewed and edited the manuscript. All authors contributed to the article and approved the submitted version.

## Funding

This study was supported by a Grant-in-Aid for Scientific Research (A) (15H01979), awarded to MH, from the Japan Society for the Promotion of Science, Tokyo, Japan. The funders had no role in the study design, data collection or analysis, decision to publish, or preparation of the manuscript.

## Conflict of Interest

The authors declare that the research was conducted in the absence of any commercial or financial relationships that could be construed as a potential conflict of interest.

## Publisher's Note

All claims expressed in this article are solely those of the authors and do not necessarily represent those of their affiliated organizations, or those of the publisher, the editors and the reviewers. Any product that may be evaluated in this article, or claim that may be made by its manufacturer, is not guaranteed or endorsed by the publisher.

## Abbreviations

PTSD: posttraumatic stress disorder
SI: suicidal ideation
DSM-5: Diagnostic and Statistical Manual for Mental Disorders
NCAM: negative alternations in cognition and mood
AAR: alterations in arousal and reactivity
PCL: PTSD checklist
CFA: confirmatory factor analysis
MDD: major depressive disorder
OCD: obsessive compulsive disorder
SAD: seasonal affective disorder
PAD: panic disorder
GAD: generalized anxiety disorder
NSSH: National Survey for Stress and Health
WHO-CIDI, World Health Organization Composite International Diagnostic Interview: version 3.0
SIDAS: Suicidal Ideation Attributes Scale.
