# Supplementary figures and images for: Associations Between PTSD Symptom Custers and Longitudinal Changes in Suicidal Ideation: Comparison Between 4-Factor and 7-Factor Models of DSM-5 PTSD Symptoms
Source: Front Psychiatry. 2021 Nov 16;12:680434. doi: 10.3389/fpsyt.2021.680434 (PMC8635060; doi:10.3389/fpsyt.2021.680434)

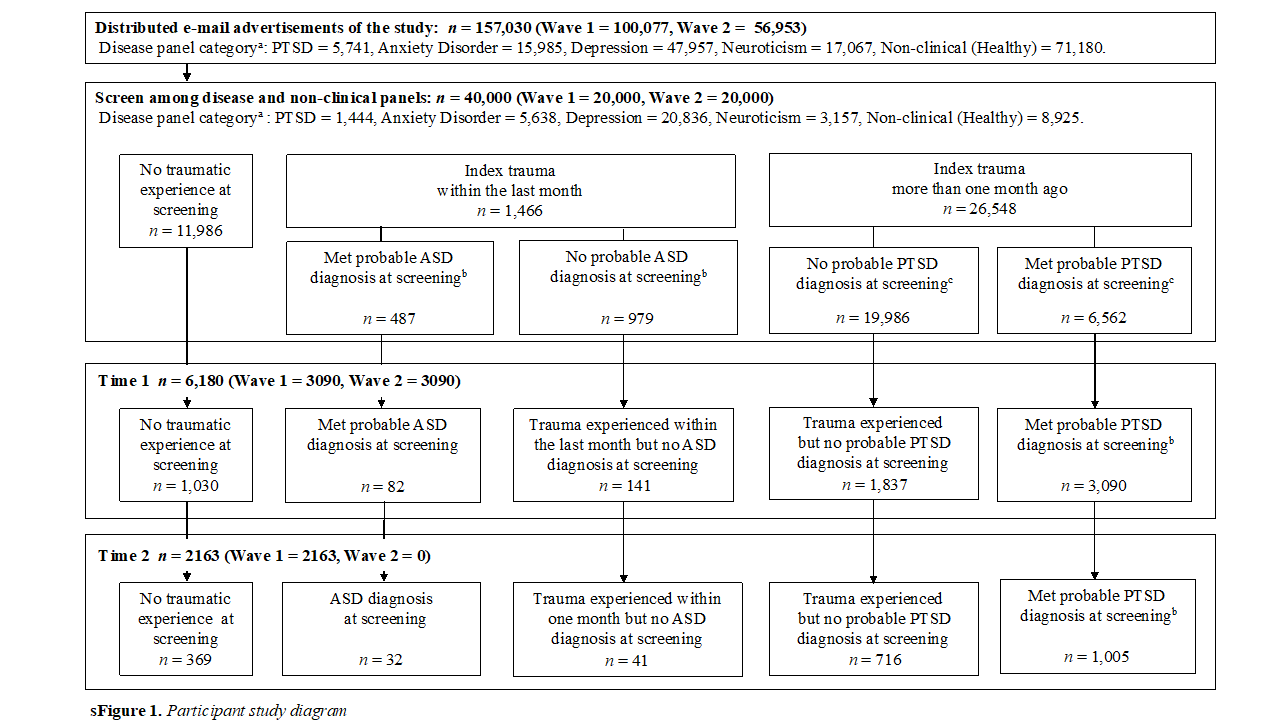

Supplement: Supplementary file 1 [file Image_1.TIF]

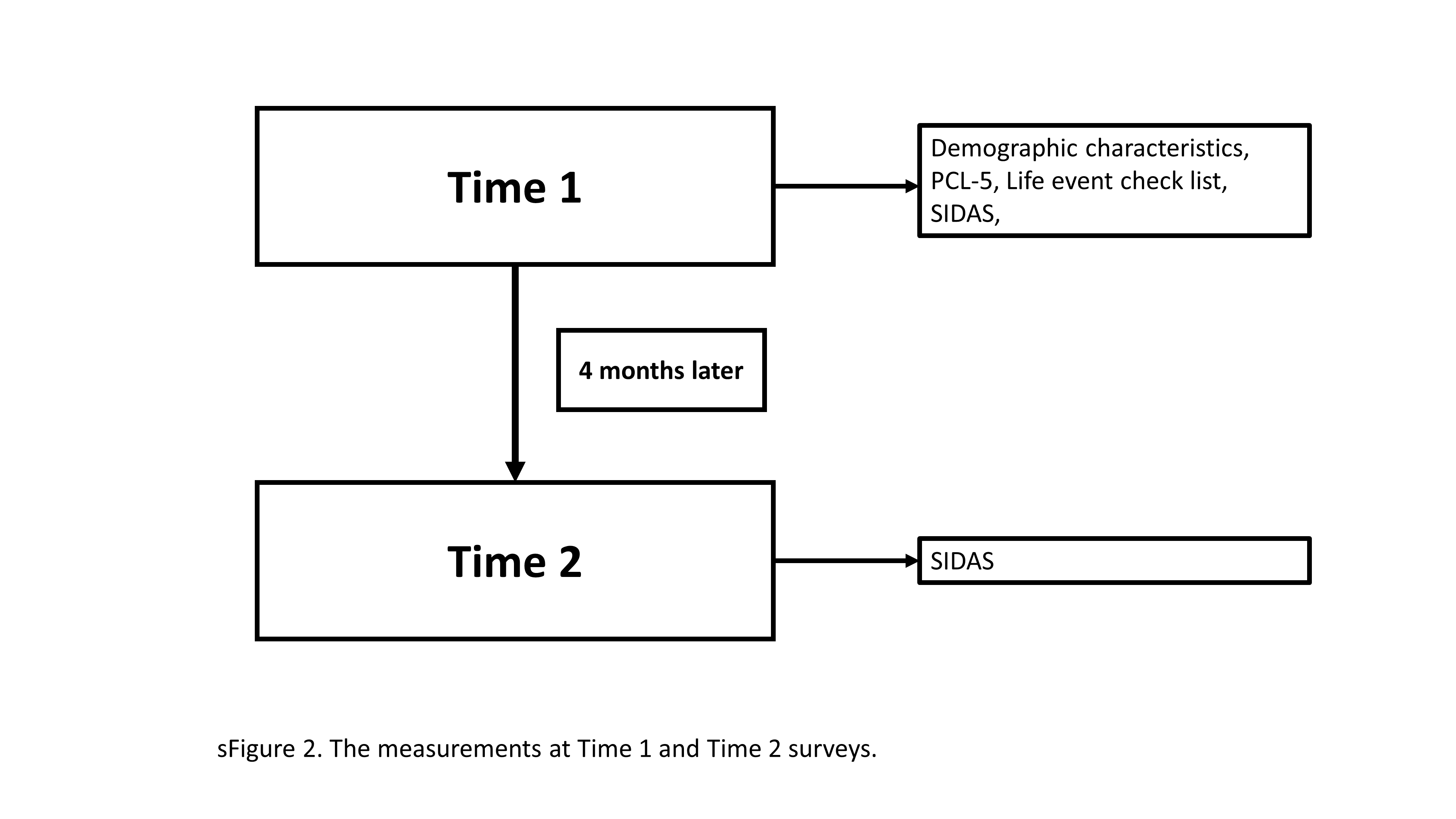

Supplement: Supplementary file 2 [file Image_2.TIF]
